# Effects of Dietary Choline Supplementation on Production Performance and Liver Characteristics in Late-Laying Hens

**DOI:** 10.3390/ani16132106

**Published:** 2026-07-07

**Authors:** Hee-Jin Kim, Hyunsoo Kim, Myunghwan Yu, Chunik Lim, Junseon Hong, Eui-Chul Hong, Yunseok Kim

**Affiliations:** Poultry Research Center, National Institute of Animal Science, Pyeongchang 25342, Republic of Korea; khj0175@korea.kr (H.-J.K.); tomymh@korea.kr (M.Y.); dlacjsdlr@korea.kr (C.L.); gospel0342@korea.kr (J.H.); drhong@korea.kr (E.-C.H.); kys88@korea.kr (Y.K.)

**Keywords:** laying hens, choline, liver, egg quality, extended laying cycle

## Abstract

This study investigated the effects of long-term dietary choline supplementation on the productive performance, liver characteristics, and egg quality of brown laying hens during an extended laying cycle from 66 to 100 weeks of age. A total of 240 Hy-Line Brown laying hens were randomly allocated to four dietary treatments containing 0%, 0.07%, 0.28%, or 0.84% supplemental Choline-50%, with six replicates per treatment. Production performance was monitored throughout the experiment, and samples for physiological analyses were collected at 100 weeks of age. Egg production during 66–75 weeks increased linearly with increasing dietary choline levels (*p* < 0.05), whereas no significant differences were observed between weeks 76 and 100. Choline supplementation significantly decreased liver fat content and malondialdehyde concentration (*p* < 0.01), indicating improved hepatic lipid metabolism and reduced oxidative stress. Small white and yellow follicle counts increased (*p* < 0.05), suggesting enhanced early follicular development. Serum inorganic phosphorus levels decreased linearly (*p* < 0.01), whereas the other blood biochemical traits were unaffected. Dietary treatments did not influence bone quality traits. Yolk weight decreased in the choline-supplemented hens (*p* < 0.05), whereas albumen quality and shell traits remained unchanged. In conclusion, dietary choline supplementation may improve liver health and maintain productivity during production cycles extending up to 100 weeks of age.

## Simple Summary

Contemporary laying hen production prolongs the laying cycle to 100 weeks to improve sustainability; however, older hens experience a decline in liver health and performance. Choline is essential for lipid metabolism; however, its long-term effects in late-laying hens remain poorly understood. This study evaluated the effects of supplemental Choline-50% (0%, 0.07%, 0.28%, and 0.84%) on Hy-Line Brown laying hens aged 66 to 100 weeks. Choline supplementation increased egg production during 66–75 weeks of age, reduced hepatic fat and oxidative stress, and increased the small follicle count, indicating improved ovarian activity. Although most egg and bone traits remained unaffected, yolk weight decreased. Overall, these results suggest that supplemental choline supports liver health and productivity throughout extended laying cycles.

## 1. Introduction

The global laying hen industry is transitioning from conventional production cycles of 72–80 weeks toward extended cycles of 90–100 weeks of age to improve efficiency and sustainability [1]. In particular, modern layer production aims to maintain high laying persistence in a single cycle without induced molting, with the long-term target of producing 500 eggs by 100 weeks of age [2]. With longer production cycles, maintaining both egg quality and hen health throughout the late laying period has become a major challenge [2].

Although genetic advancements have improved laying persistence, age-related declines in productivity and egg quality continue to be influenced by various factors, including nutrition, disease status, and management conditions [3]. Therefore, nutritional strategies are essential to sustain egg production and quality in aged hens [4]. Ensuring an adequate nutrient supply is particularly important because egg formation requires continuous metabolic conversion and nutrient transport before their deposition into the egg [2].

The liver is an important metabolic organ in laying hens and is closely associated with productive performance [5]. It is involved in the metabolism of carbohydrates, proteins, and lipids, bile production, immune regulation, and detoxification [6]. Additionally, the liver synthesizes lipoproteins required for follicle growth and yolk formation, making it essential for normal egg production [4,6]. Therefore, maintaining proper hepatic function is important for sustaining egg production and quality throughout the laying period.

The prevalence of metabolic disorders associated with excessive lipid deposition in the liver increases with advancing age [5]. Increased hepatic fat accumulation and fatty liver-related conditions may impair liver function, resulting in reduced laying performance, poorer egg quality, and weakened health status [5,7]. Consequently, nutritional approaches that maintain optimal liver health are of increasing interest, particularly for hens managed under extended production cycles.

Choline is an essential nutrient that has traditionally been grouped with the B-complex vitamins and plays a major role in lipid metabolism [8]. It is a precursor of phosphatidylcholine, a key structural phospholipid found in the liver, blood, and egg yolk, and is involved in the transport of lipids from the liver [9]. Supplemental choline has been shown to reduce hepatic lipid accumulation and improve fatty liver conditions in laying hens [10,11]. The beneficial effects of choline on hepatic lipid metabolism have also been described in other species, including humans [12].

Although birds can synthesize choline endogenously, the amount produced may not be sufficient to meet their physiological demands [12]. Poultry have a relatively limited capacity for de novo choline synthesis, and methionine or other methyl donors alone may not completely compensate for this requirement [9]. Dietary choline supplementation in laying hens can be provided in the form of chemically synthesized choline chloride or herbal choline sources, both of which have been used to support choline requirements [12]. Therefore, dietary supplementation with choline may be particularly valuable for older hens that experience increased metabolic stress.

Previous studies have reported that dietary choline supplementation can influence laying performance, hepatic lipid metabolism, plasma biochemical parameters, and egg quality in laying hens [10,11,13,14,15,16,17,18]. However, most previous studies have evaluated laying hens only during the peak and mid-laying periods (approximately 25–72 weeks of age), and studies investigating the effects of dietary choline supplementation throughout an extended laying period up to 100 weeks of age are lacking. Despite these potential benefits, information regarding the long-term effects of choline supplementation on production performance, liver condition, and egg quality in hens up to 100 weeks of age remains limited. Therefore, in this study, we investigated the effects of choline supplementation on productive performance, liver characteristics, and egg quality of brown laying hens aged 66–100 weeks.

## 2. Materials and Methods

### 2.1. Ethics Statement

The experimental protocols were reviewed and approved by the Institutional Animal Care and Welfare Committee of the National Institute of Animal Science, Rural Development Administration of the Republic of Korea (NIAS2023-0601).

### 2.2. Experimental Design and Animal Management

A total of 240 Hy-Line Brown laying hens, 66 weeks of age, were used in this study and randomly assigned to four dietary treatments with different supplemental choline concentrations. Each treatment consisted of six replicates, with each replicate comprising two adjacent cages (60 × 60 × 45 cm; length × width × height). Five hens were housed per cage, and two adjacent cages were grouped as a single replicate unit (10 hens per replicate). The experimental diets were formulated to contain 0, 0.07, 0.28, or 0.84% supplemental liquid choline chloride (liq. Choline-50%; Solton Biochem Co., Ltd., Seoul, Republic of Korea). This liquid choline supplementation was incorporated into the diets as a direct substitute for corn. All diets were based on corn and soybean meal and formulated to meet the nutrient recommendations of Hy-Line International proposed in 2020 for all nutrients except choline. The experimental diets were fed continuously throughout the trial period from 66 to 100 weeks of age. The ingredients and nutrient compositions of the experimental diets are listed in Table 1. The birds were housed in a three-tier battery cage system in an environmentally controlled, windowless facility. Feed and water were provided ad libitum throughout the experiment. Room temperature was maintained at 22 ± 3 °C, and lighting and ventilation programs were automatically controlled according to standard management practices.

### 2.3. Production Performance

Performance parameters measured during the experiment included feed intake (g/bird/day), egg production (%, hen-day basis), egg weight (g), egg mass (g/hen/day), and feed conversion ratio (kg feed/kg egg mass). Egg production was recorded daily, and feed intake was measured for each replicate. Egg mass was calculated as the product of egg production and the average egg weight. The feed conversion ratio was calculated by dividing feed intake by egg mass.

### 2.4. Sample Collection

At the end of the feeding trial, 15 hens per treatment were randomly selected and weighed. The selected hens were euthanized via carbon dioxide inhalation, and the liver, abdominal fat, oviduct, ovarian follicles, bone, and blood samples were collected. Blood samples were centrifuged to obtain the serum. Liver, bone, and serum samples were stored at −20 °C until further analysis.

### 2.5. Biochemical Analysis

Serum samples were obtained by centrifugation at 3000× *g* for 10 min at 25 °C and stored at −15 °C until analysis. Serum concentrations of total cholesterol, triglycerides, glucose, total protein, albumin, inorganic phosphorus, and calcium, as well as the activities of aspartate aminotransferase (AST) and alanine aminotransferase (ALT), were determined using an ADVIA 1650 Chemistry System (Bayer Diagnostic, Puteaux, France).

### 2.6. Organ Weights and Follicle Number

Abdominal fat was evaluated as the total weight of the fat pad surrounding the abdominal cavity and adipose tissue attached to the gizzard. Following necropsy, the weights of the liver, ovaries, and oviducts were recorded. For ovarian assessment, the ovary was excised, and follicles were classified by diameter into small white follicles (SWF, 1–3 mm), large white follicles (LWF, 3–5 mm), small yellow follicles (SYF, 5–10 mm), and large yellow follicles (LYF, >10 mm). The number of follicles in each size category was counted for each hen. Preovulatory follicles were ranked from the largest (F1) to the fifth largest (F5), and each was individually weighed using a digital balance (±0.01 g). Follicle diameter was measured for morphological evaluation.

### 2.7. Liver Characteristics

Liver fat content was determined by dual-energy X-ray absorptiometry (DEXA) using an InAlyzer system (Medikors Inc., Seongnam, Republic of Korea). Scanned images were analyzed using InAlyzer software (version 3.2.3) in high-resolution medium-scan mode. Liver fat content is expressed as a percentage of total tissue mass (%).

Liver tissues were homogenized in ice-cold extraction buffer containing 1 M Tris-HCl (Sigma-Aldrich, St. Louis, MO, USA), 5 M NaCl (Sigma-Aldrich, St. Louis, MO, USA), and 1% Triton X-100 (Sigma-Aldrich, St. Louis, MO, USA) (pH 8.0), supplemented with a protease inhibitor cocktail (GenDEPOT, Barker, TX, USA). The homogenized samples were centrifuged at 12,000× *g* for 10 min at 4 °C, and the clear supernatants were collected for subsequent assays. Total protein concentration was determined using a bicinchoninic acid (BCA) protein assay kit (Sigma-Aldrich, St. Louis, MO, USA). To determine lipid peroxidation, aliquots of the supernatant (25 μL) were treated with 30% trichloroacetic acid, 5 M HCl, or 2% thiobarbituric acid. The reaction mixtures were incubated at 90 °C for 30 min, cooled, and centrifuged again at 12,000× *g* for 10 min at 4 °C. The absorbance of the final supernatant was measured at 532 nm using a spectrophotometer. Malondialdehyde (MDA) levels were quantified using a calibration curve generated from MDA standards (Sigma-Aldrich) and expressed as nmol/mg protein.

Liver color intensity (CIE L*, a*, b*) was determined using a colorimeter (CR301 Chromameter; Minolta, Osaka, Japan). The instrument was calibrated before measurement using a white standard plate (Y = 92.40, x = 0.3136, y = 0.3196), and the color values were recorded from the liver surface.

### 2.8. Bone Characteristics

At the end of the trial, both tibiae were collected from all hens for bone quality assessment. Morphological and mechanical measurements of the left tibia, including bone weight, length, breaking strength, and cortical thickness, and those of the right tibia were used for mineral analysis. All bone samples were stored at −80 °C until evaluation and thawed at 4 °C for 24 h before analysis.

Tibial weight was measured using a precision balance (Mettler Toledo Inc., Columbus, OH, USA), and bone length was determined using a digital caliper (Mitutoyo Corp., Kanagawa, Japan; 0.01 mm accuracy). The bone breaking strength was assessed using a texture analyzer (TA1; Lloyd Instruments, Fareham, UK). Each tibia was placed with the shaft centered on the support points, and force was applied to the midpoint at a crosshead speed of 10 mm/min using a 50 kg load cell until fracture. The peak force recorded at breakage was considered the tibial breaking strength.

The bone mineral content (BMC) and bone mineral density (BMD) of the right tibia were determined by dual-energy X-ray absorptiometry (DEXA) using an InAlyzer system (Medikors Inc., Seongnam, Republic of Korea). Measurements were analyzed using the InAlyzer software (version 3.2.3) in high-resolution medium-scan mode. Bone mineral content is expressed in grams (g), whereas bone mineral density is reported as g/cm^2^.

### 2.9. Egg Quality Analysis

Thirty eggs from each replicate were randomly collected during the last week of the experiment for egg quality evaluation. Egg weight, Haugh unit, albumen height, and yolk color were determined using an EMT-5200 egg multitester (Tohoku Rhythm Co., Ltd., Saitama, Japan). Eggshell breaking strength was measured using an eggshell force gauge (Model II, Robotmation Co., Ltd., Tokyo, Japan) and expressed as the force required per unit shell surface area (kg/cm^2^). Eggshell thickness was measured at three locations (air cell, equator, and sharp end) using a digital thickness gauge (M-547; Mitutoyo Co., Ltd., Kawasaki, Japan), and the average of the three measurements was used for analysis.

### 2.10. Statistical Analysis

All data were analyzed using the General Linear Model (GLM) procedure in SAS software (version 9.4; SAS Institute Inc., Cary, NC, USA). Orthogonal polynomial contrasts were used to evaluate the linear and quadratic responses to increasing levels of dietary choline. When treatment effects were significant, mean comparisons among treatments were conducted using Duncan’s multiple-range test. Statistical significance was set at *p* < 0.05.

## 3. Results

### 3.1. Production Performance

The effects of dietary choline levels on the production performance of laying hens during the late laying period are presented in Table 2. Egg production in layer 4 (66–75 weeks) increased linearly (*p* < 0.05) with increasing dietary choline levels. Hens fed the highest choline level (0.84%) exhibited the greatest egg production (90.13%). Dietary choline levels did not significantly affect egg weight, egg mass, feed intake, or the feed conversion ratio (FCR) (*p* > 0.05). Egg production in layer 5 (76–100 weeks) increased numerically with increasing dietary choline levels; however, the differences were not significant (*p* > 0.05). Egg weight showed a significant quadratic response (*p* < 0.05), whereas other performance parameters, including egg mass, feed intake, and FCR, were not significantly affected.

### 3.2. Serum Biochemical Parameters

Serum biochemical parameters of laying hens at 100 weeks of age as affected by dietary choline levels are presented in Table 3. No significant differences in total cholesterol, triglycerides, glucose, total protein, AST, ALT, albumin, or calcium levels were observed among the dietary choline levels (*p* > 0.05). However, serum inorganic phosphorus concentration decreased linearly with increasing dietary choline levels (*p* < 0.01).

### 3.3. Reproductive Organs and Follicle Characteristics

The effects of dietary choline levels on abdominal fat, oviduct weight, and follicular characteristics are presented in Table 4 and Table 5. Abdominal fat weight was not significantly affected by dietary choline. However, oviduct weight was significantly reduced in the choline-supplemented groups compared to the control (0%) group (*p* < 0.05), exhibiting both linear (*p* < 0.05) and quadratic (*p* < 0.05) effects. Follicle weight was not affected by dietary choline levels in the F1, F4, and F5 groups (*p* > 0.05). However, the weight of F2 follicles showed a significant linear response (*p* < 0.01), whereas the weight of F3 follicles showed a significant treatment effect (*p* < 0.01) and a quadratic response (*p* < 0.05).

Dietary choline levels significantly affected the number of SWF and SYF (*p* < 0.05), whereas no significant effects were observed for LWF and LYF (*p* > 0.05). The number of SYF and SWF increased linearly with increasing dietary choline levels (*p* < 0.01), whereas LYF exhibited a quadratic response (*p* < 0.05). Regarding follicle diameter, F1 showed a significant linear effect (*p* < 0.05), whereas F2 and F3 exhibited significant quadratic responses (*p* < 0.01 and *p* < 0.05, respectively). The diameters of the remaining follicles were not significantly affected by dietary choline levels (*p* > 0.05).

### 3.4. Liver Characteristics

The effects of dietary choline levels on liver characteristics are presented in Table 6. Liver weight decreased linearly with increasing dietary choline levels (*p* < 0.05). Liver fat content was significantly reduced with increasing choline levels (*p* < 0.001), exhibiting both linear (*p* < 0.01) and quadratic (*p* < 0.01) effects (Table 6).

Additionally, the MDA concentration was significantly lower in the choline-supplemented groups than in the control (0% choline) group (*p* < 0.01) and decreased linearly with increasing dietary choline levels (*p* < 0.01). This suggests that lipid peroxidation was reduced by choline supplementation. Liver color parameters were also affected by choline supplementation; lightness (L*) and yellowness (b*) significantly decreased with increasing choline levels (*p* < 0.01), whereas redness (a*) was not significantly affected.

### 3.5. Bone Characteristics

The effects of dietary choline levels on bone characteristics are presented in Table 7. Bone weight, length, density, and strength were not significantly affected by dietary choline levels (*p* > 0.05).

### 3.6. Egg Quality

The effects of dietary choline levels on egg quality parameters are presented in Table 8. Yolk weight was significantly lower in the choline-supplemented groups than in the control (0% choline) group (*p* < 0.05). However, dietary choline levels did not affect yolk color, albumen height, Haugh unit, shell strength, or shell thickness (*p* > 0.05). Among the eggshell color parameters, a* showed a significant quadratic response (*p* < 0.05), whereas L* and yellowness b* were not significantly affected.

## 4. Discussion

The results of the present study indicated that increasing dietary choline levels (0, 0.07, 0.28, and 0.84%) did not produce consistent linear or quadratic responses in production performance, although certain measured variables were affected.

Throughout the experimental period, egg production remained within the recommended range specified in the 2020 Hy-Line Management Guide. The recommended dietary choline concentration for layer 4 and layer 5 diets is approximately 1100–1400 mg/kg, depending on feed intake and production phase. In the present study, calculated total choline concentrations ranged from 885.62 to 4527.02 mg/kg in the layer 4 diets and from 859.28 to 4500.68 mg/kg in the layer 5 diets.

In layer 4 (weeks 66–75), egg production increased linearly from 73.80% to 90.13% as dietary choline levels increased from 0 to 0.84%. In layer 5 (weeks 76–100), egg production did not differ significantly among treatments, although the 0.84% choline group showed a numerically higher value. This response differs from that reported by Zhai et al. [13], who observed a linear decrease in egg production with increasing dietary choline levels during 20–58 weeks of age, whereas no significant differences were observed during 59–68 weeks of age. The present results, indicating that dietary choline supplementation did not affect feed intake, are consistent with the findings of Dänicke et al. [14], Rajalekshmy [15], and Zhai et al. [13]. Crawford et al. [16] observed no significant change in egg production over a wide range of dietary choline levels (595–5729 mg/kg). Similarly, Lucas et al. [17] showed that neither low (300 mg/kg) nor high (2100 mg/kg) dietary choline levels affected egg production or body weight in breeder hens. These results suggest that choline supplementation in excess of adequate levels may have a limited influence on laying performance. In contrast to the present findings, Lim et al. [18] demonstrated that hens fed a choline-deficient diet exhibited significant reductions in egg production, egg mass, and feed intake, along with increased FCR, whereas choline supplementation notably improved productive performance.

The concentrations of blood metabolites, including total cholesterol, triglycerides, glucose, total protein, albumin, inorganic phosphorus, and calcium, were measured in this study, as these parameters are commonly used to evaluate metabolic status related to lipid, protein, and mineral metabolism. Blood biochemical parameters, including aspartate aminotransferase (AST) and alanine aminotransferase (ALT), are useful indicators of internal physiological status and liver function in response to dietary treatments [19]. However, when liver tissue is damaged, these enzymes are released into the circulation, resulting in elevated blood levels compared to those in healthy hens [20]. In the present study, dietary choline supplementation had a limited effect on lipid-related parameters (total cholesterol and triglycerides). Similarly, Dong et al. [12] observed no significant differences in serum triglyceride and cholesterol concentrations in hens fed diets containing 0–6800 mg/kg choline at 68 weeks of age. Daghir et al. [21] also reported that dietary choline did not influence serum cholesterol concentrations in laying hens. Additionally, serum AST and ALT activities were not significantly affected by dietary choline levels.

However, serum phosphorus concentration decreased with increasing dietary choline levels. Although choline is involved in phospholipid metabolism and phosphatidylcholine synthesis [22], the mechanism underlying the reduction in serum phosphorus remains unclear and warrants further investigation.

Ovarian follicles are fundamental functional units of the female reproductive system, and their coordinated development is essential for egg production [23]. Folliculogenesis consists of sequential processes, including follicle recruitment, selection, and maturation [24]. Ovarian follicles are classified into prehierarchical and hierarchical groups according to developmental stage. Prehierarchical follicles include SWF, LWF, SYF, and LYF, whereas selected hierarchical follicles are ranked from F1 to F5 or F6, with F1 being the largest preovulatory follicle [24]. Follicle size and number are commonly used as practical indicators of reproductive status [25]. These findings partially support previous observations regarding the role of choline in follicular development. In particular, the increase in the numbers of SWF and SYF observed in this study suggests that choline supplementation may influence early-stage folliculogenesis. Similarly, Liu et al. [25] reported that dietary supplementation with 600 mg/kg choline significantly increased the number of SWF in ducks during the late laying period. These results suggest that choline may support ovarian activity by promoting the recruitment and development of prehierarchical follicles, possibly through its role in phosphatidylcholine synthesis, a major component of cell membranes [26]. Phosphatidylcholine is important for rapidly proliferating follicular cells, and impaired phosphatidylcholine biosynthesis has been associated with disrupted follicular development and oocyte maturation [27,28]. Thus, dietary choline may contribute to early follicular growth through its involvement in membrane formation and lipid metabolism.

The present study demonstrated that dietary choline supplementation significantly affected hepatic lipid metabolism and oxidative status in laying hens at 100 weeks of age. In particular, liver fat content decreased with increasing dietary choline levels, showing both linear and quadratic responses. These findings are consistent with previous studies indicating that choline supplementation reduces hepatic lipid accumulation in laying hens [12,29]. Specifically, Dong et al. [12] demonstrated that increasing dietary choline levels (0–6800 mg/kg) significantly decreased liver lipid accumulation during the laying period. In laying hens, the liver plays a central role in the synthesis and transport of lipids for yolk formation, making it highly susceptible to lipid accumulation and metabolic disorders, such as fatty liver hemorrhagic syndrome [11]. Therefore, the reduced hepatic lipid content observed in the present study suggests that dietary choline supplementation may support liver function and metabolic stability, consistent with previous studies in laying hens and broilers [30]. Although liver weight was not significantly affected, its decreasing tendency suggests a potential reduction in hepatic lipid accumulation.

In addition to lipid metabolism, choline supplementation also affected hepatic oxidative status, as indicated by the significant reduction in MDA concentration. MDA is a widely recognized marker of lipid peroxidation, and its decrease indicates reduced oxidative stress in the liver. Similar findings have been reported by Yonke and Cherian [31], who observed decreased hepatic MDA levels in laying hens after choline supplementation.

The antioxidant effects of choline may be related to its role as a methyl donor in one-carbon metabolism, which supports glutathione synthesis and helps maintain cellular antioxidant capacity [8,32]. Consistent with previous findings, Aziza et al. [8] reported increased hepatic α-tocopherol concentrations following choline supplementation, suggesting that dietary choline may contribute to improved hepatic antioxidant status in late-laying hens.

Additionally, liver color parameters were significantly affected by dietary choline levels, particularly the L* and b* values, which decreased as choline levels increased. These changes may indicate alterations in hepatic lipid content and metabolic status, as liver color is typically associated with fat accumulation and tissue composition. The reduction in L* and b* observed in the present study was consistent with decreased lipid deposition in the liver. As liver color can be visually assessed, it may serve as a practical preliminary indicator of hepatic fat status in commercial laying hens. Overall, these findings indicate that dietary choline plays a critical role in improving the liver health of late-laying hens by reducing lipid accumulation and oxidative stress.

During the late laying period, bone health is increasingly compromised because structural bone is progressively depleted to support eggshell formation, frequently leading to osteoporosis and associated welfare and economic concerns [33]. Therefore, maintaining bone integrity is essential because it is closely associated with both egg production performance and eggshell quality [34]. Reduced bone strength in laying hens is associated with a decline in productivity and shell quality [34]. However, in the present study, dietary choline supplementation did not have any significant effect on bone-related parameters.

The Haugh unit is a widely accepted standard indicator of albumen quality and egg freshness. In the present study, the Haugh unit values were not significantly altered by increasing dietary choline levels, although numerically higher values were observed in the 2800 and 8400 mg/kg groups than in the control. These findings are consistent with those of Tsiagbe et al. [35], who reported that supplementation with 500 or 1000 mg/kg choline did not affect the Haugh unit scores in hens at 44 and 64 weeks of age compared to a basal diet containing 1040 mg/kg choline. Similarly, Daghir et al. [21] found no effect of dietary choline (889–2222 mg/kg) on Haugh units. In contrast, Zhai et al. [13] observed improved Haugh unit values when hens were supplemented with 425–6800 mg/kg choline at 59–68 weeks of age. These inconsistent responses among studies may be attributed to differences in hen age, basal dietary choline concentrations, production stages, or environmental and management conditions. The hens in the present study may have already received sufficient choline from the basal diet, thereby limiting any additional responses to supplementation.

Egg production is closely related to ovarian follicular development, and hierarchical follicle growth requires continuous deposition of yolk precursors synthesized in the liver and transported to the ovary [36,37]. Therefore, the numerical increase in egg production and ovulation rate, together with improved hepatic lipid mobilization, may have reduced lipid deposition per individual yolk, thereby contributing to the decreased yolk weight. However, further studies are required to elucidate the precise mechanisms underlying these responses.

The yolk color score was not significantly influenced by dietary choline supplementation, which is consistent with the findings of Zhai et al. [13], who reported no effect of 0–6800 mg/kg choline supplementation on yolk pigmentation in hens from 59 to 68 weeks of age. However, Dänicke et al. [14] demonstrated that supplementation with 1000 mg/kg choline significantly decreased yolk pigmentation, whereas 4000 mg/kg choline had no effect, suggesting that different choline inclusion levels may influence pigment deposition through various metabolic pathways. The mechanisms underlying choline metabolism and yolk pigment dynamics remain unclear and require further investigation.

A potential concern associated with high dietary levels of choline chloride is the development of a fishy odor in eggs, mainly through the accumulation of trimethylamine [38]. Egg odor and trimethylamine concentration were not measured in the present study; therefore, the occurrence of a fishy odor could not be directly evaluated. Although no sensory abnormality was recorded during routine egg quality assessment, further studies including trimethylamine analysis and sensory evaluation are needed to determine whether high choline chloride supplementation affects egg odor in late-laying hens.

Shell quality parameters, including shell strength and thickness, were not significantly affected by choline supplementation. This result aligns with the findings of Zhai et al. [13], who observed no effect of dietary choline (0–6800 mg/kg) on shell strength, and Daghir et al. [21], who revealed no changes in shell quality traits with supplemental choline. Although eggshell strength is economically important for reducing cracked or broken eggs [39], the present results indicate that choline supplementation does not directly contribute to shell structural improvement in late-laying hens.

Overall, our findings suggest that dietary choline supplementation has minimal effects on most egg quality parameters in late-laying hens, indicating that supplemental choline may be more closely associated with metabolic health and production responses than with direct improvements in egg quality characteristics.

## 5. Conclusions

Dietary choline supplementation had limited effects on the overall production performance and egg quality of brown laying hens aged 66–100 weeks. Although choline supplementation improved several indicators of liver health by reducing hepatic fat accumulation and lipid peroxidation, its effects on productivity were minimal. Choline supplementation partially enhanced follicular development and early late-laying egg production. These findings suggest that supplemental choline may be a useful nutritional strategy to support metabolic stability and liver function in aged laying hens, rather than notably improving productivity. This study is meaningful because the hens were maintained up to 100 weeks of age, whereas relatively few studies have evaluated the effects of dietary interventions throughout such an extended laying period. Further studies investigating lipid transport and related metabolic pathways are needed to better understand the role of choline in supporting liver function and metabolic stability in late-laying hens.

## Figures and Tables

**Table 1:** Ingredient composition and calculated nutrient content of experimental diets fed to Hy-Line Brown laying hens supplemented with four dietary choline levels during the late laying period.

	Layer 4 (66–75 Weeks)				Layer 5 (76–100 Weeks)			
Dietary Choline-50% (%)	0	0.07	0.28	0.84	0	0.07	0.28	0.84
Corn, %	62.94	62.87	62.66	62.10	63.61	63.54	63.33	62.77
Soybean meal, %	19.82	19.82	19.82	19.82	18.78	18.78	18.78	18.78
Animal Fat, %	3.31	3.31	3.31	3.31	3.45	3.45	3.45	3.45
Limestone, %	9.70	9.70	9.70	9.70	10.08	10.08	10.08	10.08
Carrier (Corn), %	1.48	1.48	1.48	1.48	1.46	1.46	1.46	1.46
Corn Gluten, %	0.16	0.16	0.16	0.16	0.15	0.15	0.15	0.15
Choline chloride-50%, %	0.00	0.07	0.28	0.84	0.00	0.07	0.28	0.84
Tricalcium Phosphate, %	1.56	1.56	1.56	1.56	1.42	1.42	1.42	1.42
NaCl, %	0.38	0.38	0.38	0.38	0.37	0.37	0.37	0.37
DL-Methionine (98%), %	0.25	0.25	0.25	0.25	0.24	0.24	0.24	0.24
L-Lysine-Sulfate (54%), %	0.11	0.11	0.11	0.11	0.15	0.15	0.15	0.15
Tryptophan-10%, %	0.01	0.01	0.01	0.01	0.01	0.01	0.01	0.01
Vitamin, %	0.14	0.14	0.14	0.14	0.14	0.14	0.14	0.14
Minerals, %	0.14	0.14	0.14	0.14	0.14	0.14	0.14	0.14
Metabolizable energy, kcal/kg	2820.45	2818.18	2811.35	2793.15	2847.36	2845.09	2838.26	2820.06
Crude protein, %	14.22	14.22	14.20	14.16	13.89	13.89	13.87	13.83
Total lysine, %	0.76	0.76	0.76	0.76	0.75	0.75	0.75	0.75
Total methionine, %	0.48	0.48	0.48	0.48	0.47	0.47	0.47	0.47
Total methionine + cysteine, %	0.72	0.72	0.72	0.72	0.70	0.70	0.70	0.70
Calcium, %	4.18	4.18	4.18	4.18	4.27	4.27	4.27	4.27
Sodium, %	0.17	0.17	0.17	0.17	0.17	0.17	0.17	0.17
Total phosphorus, %	0.32	0.32	0.32	0.32	0.29	0.29	0.29	0.29
Choline, mg/kg	885.62	1189.07	2099.42	4527.02	859.28	1162.73	2073.08	4500.68

Nutrient values are calculated based on ingredient composition. Betaine content and analyzed nutrient values were not independently determined in the present study.

**Table 2:** Production performance of laying hens during the late laying period (66–100 weeks of age) as affected by dietary choline levels.

Parameters	Dietary Choline-50% (%)				SEM ^1^	*p*-Value		
	0	0.07	0.28	0.84		Treatment	Linear	Quadratic
Layer 4 (66–75 weeks)								
Egg production (%)	73.80	79.69	82.79	90.13	4.527	0.121	0.027	0.441
Egg weight (g)	64.87	65.15	65.53	65.70	0.891	0.911	0.818	0.969
Egg mass	48.05	51.98	54.17	59.35	3.310	0.151	0.101	0.568
Feed intake (g)	122.88	113.58	124.36	128.94	8.550	0.644	0.513	0.300
FCR	2.60	2.22	2.32	2.18	0.162	0.290	0.201	0.286
Layer 5 (76–100 weeks)								
Egg production (%)	72.45	73.28	78.16	85.02	3.782	0.126	0.107	0.241
Egg weight (g)	66.15	67.17	65.34	66.23	1.518	0.866	0.732	0.042
Egg mass	48.13	49.25	51.28	56.35	3.364	0.361	0.153	0.122
Feed intake (g)	103.44	115.25	110.90	98.46	15.356	0.662	0.795	0.102
FCR	2.19	2.34	2.16	1.65	0.297	0.415	0.196	0.050

^1^ SEM, standard error of the mean; FCR, feed conversion ratio.

**Table 3:** Serum biochemical parameters of laying hens at 100 weeks of age as affected by dietary choline levels.

Parameters	Dietary Choline-50% (%)				SEM ^1^	*p*-Value		
	0	0.07	0.28	0.84		Treatment	Linear	Quadratic
Total cholesterol (mg/dL)	147.41	136.37	147.90	148.89	8.559	0.742	0.863	0.510
Triglyceride (mg/dL)	1152.70	976.01	963.41	1054.76	97.753	0.494	0.525	0.176
Glucose (mg/dL)	147.29	155.40	134.29	136.87	10.210	0.490	0.837	0.799
Total protein (g/dL)	6.38	5.93	6.15	5.95	0.169	0.282	0.074	0.481
AST (U/L)	194.30	206.09	197.74	237.86	23.151	0.588	0.219	0.566
ALT (U/L)	4.02	3.15	4.54	3.32	0.991	0.758	0.116	0.534
Albumin (g/dL)	2.32	2.19	2.30	2.32	0.037	0.102	0.548	0.082
Inorganic phosphorus (mg/dL)	6.59	5.44	5.89	4.98	0.322	0.170	0.001	0.694
Calcium (mg/dL)	29.70	25.72	29.01	28.86	1.208	0.116	0.164	0.199

^1^ SEM, standard error of the mean.

**Table 4:** Abdominal fat, oviduct, and follicle weights of laying hens at 100 weeks of age as affected by dietary choline levels (*n* = 8).

Parameters		Dietary Choline-50% (%)				SEM ^1^	*p*-Value		
		0	0.07	0.28	0.84		Treatment	Linear	Quadratic
Abdominal fat (g)		103.18	112.63	104.83	77.06	17.959	0.536	0.293	0.309
Oviduct weight (g)		82.26 ^a^	70.11 ^b^	69.95 ^b^	71.36 ^b^	2.949	0.017	0.019	0.029
Follicle weight (g)	F1	16.08	14.65	14.17	14.47	0.094	0.298	0.117	0.250
	F2	8.36	5.89	10.61	11.19	0.733	0.284	0.001	0.071
	F3	8.36 ^a^	5.89 ^b^	6.23 ^ab^	7.53 ^ab^	0.812	0.001	0.533	0.020
	F4	4.24	2.82	3.42	4.13	0.761	0.102	0.927	0.122
	F5	1.76	1.17	1.68	1.86	0.668	0.414	0.590	0.265

^1^ SEM, standard error of the mean. ^a,b^ Means within a row with different superscripts differ significantly (*p* < 0.05).

**Table 5:** Follicle number and diameter of laying hens at 100 weeks of age as affected by dietary choline levels (*n* = 8).

Parameters		Dietary Choline-50% (%)				SEM ^1^	*p*-Value		
		0	0.07	0.28	0.84		Treatment	Linear	Quadratic
Follicle number	SWF	36.30 ^b^	34.88 ^b^	32.38 ^b^	48.00 ^a^	3.650	0.025	0.049	0.029
	LWF	19.50	19.13	17.50	16.00	1.990	0.588	0.184	0.779
	SYF	13.25 ^b^	12.13 ^b^	16.75 ^ab^	19.00 ^a^	1.724	0.032	0.008	0.336
	LYF	5.25	4.63	5.00	5.38	0.243	0.161	0.496	0.049
Follicle diameter (mm)	F1	3.36	3.13	3.14	3.14	0.243	0.161	0.044	0.089
	F2	3.06	2.63	2.79	2.85	0.067	0.058	0.222	0.006
	F3	2.66	2.16	2.34	2.45	0.085	0.051	0.431	0.019
	F4	2.04	1.75	1.86	1.99	0.123	0.052	0.967	0.120
	F5	1.49	1.28	1.48	1.50	0.132	0.415	0.581	0.227

^1^ SEM, standard error of the mean; SWF, small white follicles; LWF, large white follicles; SYF, small yellow follicles; LYF, large yellow follicles. ^a,b^ Means within a row with different superscripts differ significantly (*p* < 0.05).

**Table 6:** Liver characteristics of laying hens at 100 weeks of age as affected by dietary choline levels.

Parameters		Dietary Choline-50% (%)				SEM ^1^	*p*-Value		
		0	0.07	0.28	0.84		Treatment	Linear	Quadratic
Liver weight (g)		51.28	53.25	57.29	42.19	3.020	0.076	0.028	0.144
Liver fat (%)		17.05 ^a^	19.56 ^a^	15.92 ^b^	11.88 ^c^	0.885	<0.001	0.006	0.001
MDA (μmol/mg protein)		108.02 ^a^	87.25 ^b^	87.66 ^b^	87.55 ^b^	4.665	0.008	0.006	0.035
Liver color	L*	50.24 ^a^	55.22 ^a^	46.50 ^b^	40.41 ^c^	1.598	<0.001	0.007	0.002
	a*	17.36	15.09	16.10	15.90	0.661	0.136	0.080	0.128
	b*	28.61 ^a^	29.99 ^a^	26.30 ^a^	18.42 ^b^	1.507	<0.001	<0.001	0.005

^1^ SEM: standard error of the mean; MDA: malondialdehyde. ^a–c^ Means within a row with different superscripts differ significantly (*p* < 0.05).

**Table 7:** Bone characteristics of laying hens at 100 weeks of age as affected by dietary choline levels.

Parameters	Dietary Choline-50% (%)				SEM ^1^	*p*-Value		
	0	0.07	0.28	0.84		Treatment	Linear	Quadratic
Bone weight (g)	13.61	13.19	13.09	12.31	0.526	0.382	0.118	0.742
Bone length (mm)	122.23	123.45	121.76	120.57	1.070	0.313	0.494	0.270
Bone density (g/cm^2^)	0.37	0.36	0.36	0.34	0.027	0.841	0.468	0.768
Bone strength (kgf)	31.37	30.92	29.61	30.43	2.957	0.978	0.909	0.830

^1^ SEM: standard error of the mean.

**Table 8:** Egg quality parameters of laying hens at 100 weeks of age as affected by dietary choline levels.

Parameters		Dietary Choline-50% (%)				SEM ^1^	*p*-Value		
		0	0.07	0.28	0.84		Treatment	Linear	Quadratic
Albumen height (mm)		6.75	6.92	7.36	7.88	0.404	0.199	0.104	0.665
Haugh units		77.55	76.16	81.68	83.39	2.975	0.277	0.370	0.604
Yolk weight (g)		18.27 ^a^	18.16 ^a^	17.43 ^b^	17.33 ^b^	0.292	0.043	0.101	0.991
Yolk color score		8.12	7.98	7.73	7.67	0.166	0.195	0.266	0.543
Shell strength (kgf)		3.33	3.72	3.42	3.64	0.198	0.460	0.162	0.679
Shell thickness (mm)		0.43	0.43	0.42	0.41	0.006	0.544	0.260	0.596
Shell color	L*	61.24	60.11	59.54	61.93	0.902	0.237	0.514	0.054
	a*	18.27	19.67	19.75	18.48	0.509	0.078	0.813	0.010
	b*	31.79	32.40	32.53	31.76	0.415	0.420	0.902	0.098

^1^ SEM: standard error of the mean. ^a,b^ Means within a row with different superscripts differ significantly (*p* < 0.05).

## Data Availability

The data presented in this study are available from the corresponding author upon reasonable request.
